# Live attenuated SARS-CoV-2 vaccine OTS-228 demonstrates efficacy, safety, and stability in preclinical model

**DOI:** 10.1038/s41541-025-01165-2

**Published:** 2025-05-23

**Authors:** Tobias Britzke, Nico Joël Halwe, Lorenz Ulrich, Angele Breithaupt, G. Tuba Barut, Claudia Wylezich, Nadine Ebert, Bettina Salome Trüeb, Volker Thiel, Donata Hoffmann, Martin Beer, Jacob Schön

**Affiliations:** 1 Department of Experimental Animal Facilities and Biorisk Management, Friedrich-Loeffler-Institut, Greifswald, Germany; 2 Institute of Diagnostic Virology, Friedrich-Loeffler-Institut, Greifswald, Germany; 3 Institute of Virology and Immunology, Vetsuisse Faculty, University of Bern, Bern and Mittelhäusern, Switzerland; 4 Department of Infectious Diseases and Pathobiology, Vetsuisse Faculty, University of Bern, Bern, Switzerland; 5 Multidisciplinary Center for Infectious Diseases, Vetsuisse Faculty, University of Bern, Bern, Switzerland; 6 European Virus Bioinformatics Center, Jena, Germany

**Keywords:** Adaptive immunity, Infectious diseases, Vaccines, Vaccines, Virology, Infectious diseases, Respiratory tract diseases, Infection

## Abstract

Live attenuated vaccines (LAV) have the potential to meet all the criteria for an efficacious vaccine. In addition to providing protection against the target disease, they offer the potential to prevent transmission, provide cross-protection by stimulating humoral and cellular immunity, and allow versatility in application routes. The SARS-CoV-2 LAV candidate, OTS-228, has demonstrated excellent safety and high efficacy in preclinical models, inducing transmission-blocking immunity and providing full protection, even against variants such as Omicron BA.2, BA.5, and XBB.1.5. However, to ensure that OTS-228 has no dose-dependent side effects and to evaluate potential risk of reversion to virulence—a known general issue with live vaccines—detailed characterization of LAV OTS-228 is essential. To address this, we conducted four different experiments using Syrian hamsters, a model for moderate to severe COVID-19. A maximum dose trial confirmed the vaccine’s full attenuation and prevention of transmission, even at high doses. In addition, four intentional serial in vivo passages demonstrated the genomic stability of the vaccine and the non-infectivity of nasal washings. Furthermore, OTS-228 maintained its attenuation and immunogenicity even after 15 additional in vitro passages, providing full protection against lung infection with virulent SARS-CoV-2 strains. Finally, a low-dose experiment confirmed the high efficacy of the vaccine candidate, establishing the protective dose 50 (PD_50_) at less than 100 TCID_50_ per hamster. Our results provide strong evidence for the safety and efficacy of the LAV candidate OTS-228 and supports its potential as a safe and effective vaccine in a highly relevant preclinical model.

## Introduction

Live vaccines may offer several important advantages over other types of vaccines, including prevention of transmission, improved cross-protection against antigenically diverse virus variants, and the potential for non-invasive delivery^1–3^. Our previously described live attenuated SARS-CoV-2 vaccine (LAV), OTS-228, has demonstrated optimal safety as it is well attenuated in preclinical animal models and does not transmit to serologically naïve contacts when administered at moderate titers^4^. Attenuation was achieved by converting 325 serine and leucine codons in the ORF1ab into so-called “one-to-stop” (OTS) codons, which are just one random mutation away from becoming stop codons, thereby placing the virus in an unfavorable evolutionary niche^4^. This was combined with two amino acid changes (K164A, H165A) in non-structural protein 1 (nsp1), the deletion of accessory open reading frames (ORFs) 6-8, and the deletion of the spike S1/S2 polybasic cleavage site (PCS) (Supplementary Figure 1a)^4^. Moreover, OTS-228 has demonstrated high efficacy by inducing sterile immunity when challenged with the antigenically matching SARS-CoV-2 wild type (WT), and provided clinical protection against various variants of concern, such as Omicron BA.2, BA.5, and XBB.1.5^4^.

LAVs are still replication-competent, and reversion to virulence has been reported for other LAVs^5,6^. Therefore, it is crucial to test the stability of attenuation and to estimate the potential for regaining virulence. Additionally, protection provided by LAVs at low dosages would be a highly beneficial feature for subsequent clinical trials and applications. To address these points, we conducted four separate experiments in Syrian hamsters, an established and sensitive preclinical model of COVID-19.

The first objective was to confirm the attenuation of OTS-228 and its lack of transmission between Syrian hamsters following administration of the possible maximum dose of 10^6.1^ TCID_50_ per hamster. Second, we evaluated the genetic stability of OTS-228 through both in vivo and in vitro passaging. In vivo, OTS-228 was intentionally passaged four times from hamster to hamster. In vitro, the OTS-228 LAV was passaged 15 times using Vero E6 cells and then used to vaccinate Syrian hamsters at the maximum feasible dose to confirm the attenuated phenotype and immunogenic profile. These hamsters were subsequently challenged with virulent SARS-CoV-2 Alpha and Delta variants to verify preserved efficacy. Finally, we determined the minimal dose of OTS-228 to induce a robust immune response and provide protection against SARS-CoV-2 WT infection.

## Results

### Intranasal administration of a maximum dose of OTS-228 in Syrian hamsters confirms attenuation and absence of transmission

**Fig. 1:**
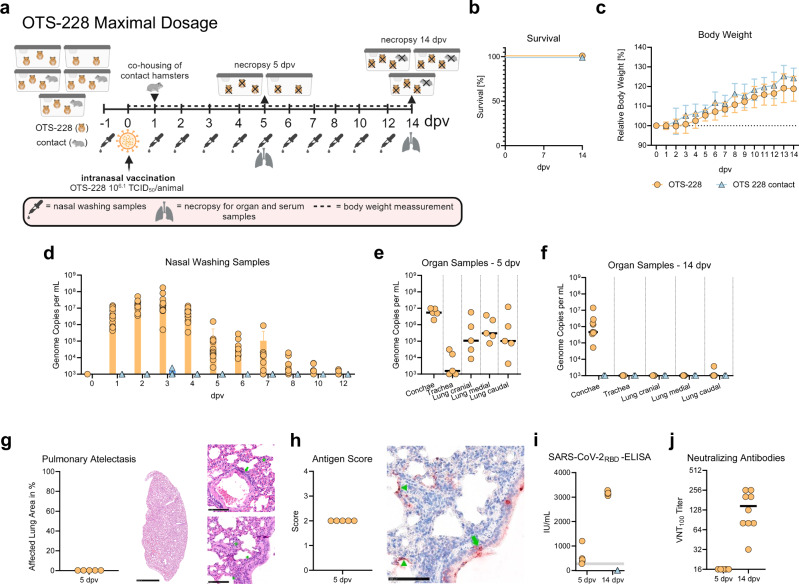
Intranasal vaccination of Syrian hamsters with a maximum dose of OTS-228 confirms attenuation and immunogenicity. **a** Experimental setup: Syrian hamsters (n = 14) were intranasally vaccinated with the maximum applicable dose of 10^6.1^ TCID_50_ of OTS-228 per animal. At 1 dpv, serologically naïve direct contact animals (n = 3) were co-housed with vaccinated animals in a 1:3 ratio to detect transmission events. **b** Survival rate and (**c**) body weight were monitored over 14 dpv, confirming no mortality and no weight loss. **d** Nasal wash samples were analyzed via RdRp (nsp12)-specific qPCR to determine genome copies per mL (gc/mL). Genome copy numbers were calculated based on a standard of known concentration. No viral genome shedding was detected in contact animals throughout the experiment. Bars indicate mean with SD. **e** Tissue sampling: Five vaccinated hamsters were euthanized at 5 dpv, and (**f**) the remaining nine vaccinated and three contact hamsters were euthanized at 14 dpv to assess genome copies in respiratory tract organ samples. At 5 dpv, virus genome was detected in nasal conchae and lung samples, but by 14 dpv, viral genome was completely cleared from lung samples and absent in contact animals. Mean is indicated by line. **g** Histopathology at 5 dpv showed no pneumonia-related atelectasis, shown as percentage of affected area. Representative hematoxylin-eosin stained sections showed the lack of atelectasis (whole slide image, scale bar 2.5 mm), along with interstitial macrophage infiltrates (green asterisk), perivascular (green arrow), and peribronchial immune cell infiltration (green arrowhead) (detailed images, scale bar 100 µm). **h** Antigen score and immunohistochemistry: Representative anti-SARS-CoV N-protein immunohistochemistry of lung sections showed multifocal virus antigen presence in type I pneumocytes (green arrowhead) and bronchial epithelial cells (green arrow) associated with the peribronchial and interstitial immune cell infiltration, shown in a consecutive slide of Fig. 1g, right lower image (scale bar 100 µm). **i** Serum antibodies specific to the SARS-CoV-2 RBD domain were detected in all vaccinated animals at 14 dpv, while absent in contact animals. **j** Virus neutralization test (VNT_100_): The sera from vaccinated animals also exhibited neutralizing capacity in a virus neutralization test.

To determine whether OTS-228 has an optimal safety profile even when administered at a maximum dose, we intranasally vaccinated Syrian hamsters (n = 14) at the highest technically feasible dose, equivalent to an infectious titer of 10^6.1^ TCID_50_ per animal, as confirmed by back titration of the inoculum. The study included the co-housing of serologically naïve direct contact animals (n = 3) one day post-vaccination (dpv), at a ratio of one naïve contact animal to three vaccinated animals (Fig. 1a). Neither the vaccinated nor the contact animals showed mortality (Fig. 1b) or significant weight loss; only 4 out of 14 vaccinated hamsters showed a slight decrease in body weight at 3 dpv compared to baseline (Fig. 1c).

Viral genome was detected in the nasal washings of vaccinated hamsters up to 12 dpv, peaking at 3 dpv. Although two 3 dpv nasal washing were infectious in cell culture (Supplementary Figure 3), the vaccine was not shed to any of the direct contact animals (Fig. 1d). At 5 dpv, the vaccine virus genome was detectable in respiratory tissues of both the upper (conchae and trachea) and lower respiratory tract (LRT, including cranial, medial, and caudal right lung lobes) (Fig. 1e). However, by 14 dpv, analysis of samples from the trachea and LRT revealed almost complete clearance of vaccine virus RNA, with detectable viral genome only in conchal samples from vaccinated animals (Fig. 1f). Importantly, none of the organ samples from contact animals tested positive for vaccine virus genome, reinforcing the absence of viral shedding to contact animals (Fig. 1f).

Histopathologic examination of the lungs at 5 dpv revealed that vaccination with the maximum dose of OTS-228 did not result in pneumonia-induced atelectasis, SARS-CoV-2 characteristic vascular lesions, or necrotizing bronchitis. However, peribronchial (5/5) and perivascular (5/5) inflammatory infiltrates were consistently observed, with some immune cells adhering to the vascular endothelium (3/5) (Fig. 1g). The detection of viral antigen (5/5) was associated with a slight expansion of the pulmonary interstitium primarily due to macrophages (Fig. 1h).

A humoral immune response was confirmed in the vaccinated animals by the SARS-CoV-2 WT RBD-specific ELISA (Fig. 1i) at 14 dpv, along with live virus neutralizing capacity (Fig. 1j). The absence of a serologic response in direct-contact animals corroborates the lack of transmission of the OTS-228 vaccine virus (Fig. 1i and j).

### OTS-228 is genetically stable and resistant to forced transfer via nasal wash specimens

**Fig. 2:**
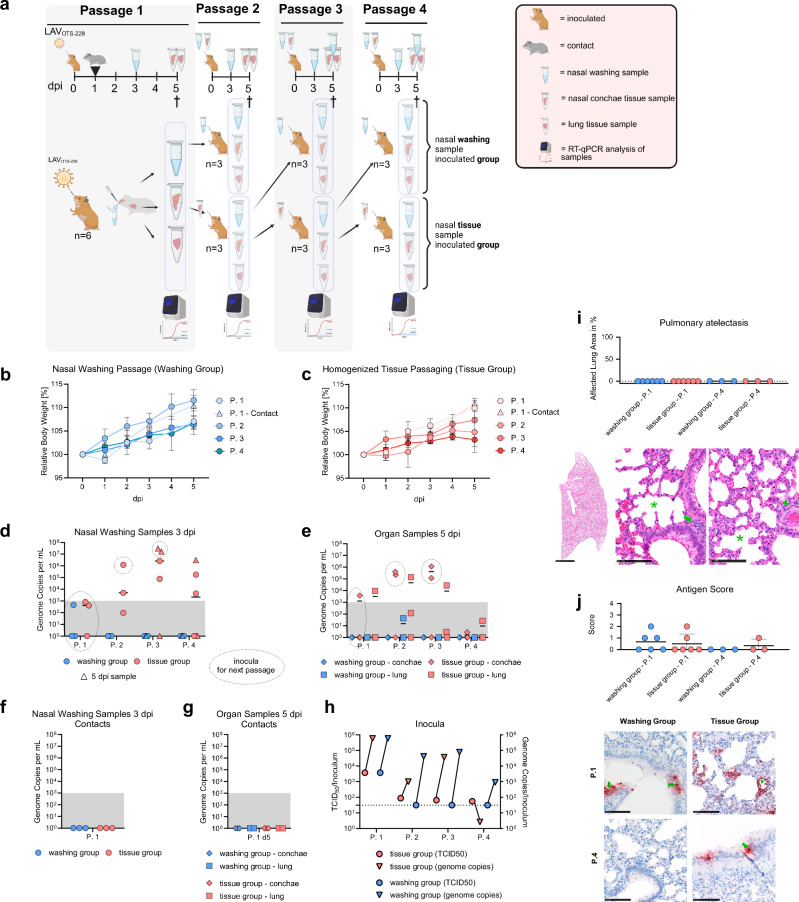
Forced in vivo passaging confirms robust attenuation of OTS-228. **a** Experimental setup: Intranasal inoculation of six index hamsters with OTS-228 (10^3.8^ TCID_50_/animal), followed by three consecutive passages using either nasal wash samples (“washing group”) or conchae samples (“tissue group”) as inoculum. **b** Body weight: None of the hamsters in the washing group or (**c**) tissue group experienced body weight loss, regardless of passage number. **d** Viral genome detection: Virus genome was found only in the nasal washing samples of the tissue group and in the (**e**) tissue samples, while the washing group remained negative throughout all passages. **f**, **g** Transmission: Direct contact animals did not become infected when co-housed with inoculated animals during passage 1. **h** Viral load: The viral quantity in the inoculum was determined by titration and RT-qPCR. **i** Histopathology: Examination of animals from passage 1 and passage 4 revealed no pneumonia-related atelectasis, shown as a percentage of the affected area. Representative hematoxylin-eosin stained sections showed no atelectasis (whole slide image, bar = 2.5 mm), no alveolar immune cell infiltration (green asterisk), but the presence of perivascular (green arrowhead) and peribronchial inflammatory infiltrates (green arrow), detailed images, bar = 100 µm. **j** Antigen detection: The same samples were screened for SARS-CoV N-protein antigen. The antigen score and representative immunohistochemistry lung sections illustrate multifocal viral antigen presence in type-I pneumocytes (green arrowhead) and bronchial epithelial cells (green arrow), bar = 100 µm.

To assess the genomic stability of OTS-228 and the preservation of its attenuated phenotype, we intentionally passaged OTS-228 four times from hamster to hamster. For the initial inoculation, an OTS-228 cell culture-derived stock was used. Since the OTS-228 vaccine virus does not naturally transmit to contact animals, subsequent passages were based either on nasal washing samples (“washing group”) or on the supernatant of homogenized nasal conchae tissue material (“tissue group”). The experimental design is shown in Fig. 2a. No sustained body weight loss was observed in any group, regardless of the passage number (Fig. 2b, c).

Nasal washings failed to induce infection. Only in the tissue group successful infection could be demonstrated by evaluation of nasal washing (Fig. 2d) and tissue samples (Fig. 2e) that were found positive for viral genome. Thus, for the nasal washing group, inoculation relied on the positive nasal washing samples from the tissue group, as indicated by circles in Fig. 2d, e. The natural block of transmission was confirmed by the absence of viral genome in nasal washing and tissue samples from direct contact animals at passage 1 (P.1) (Fig. 2f, g).

Although the inocula from the tissue and washing groups were comparable in terms of viral genome copies, no in vitro infectivity was detected in the washing samples (Fig. 2h). Histopathological examination of the lungs from animals in the initial (P.1) and final (P.4) passages did not reveal pneumonia-induced atelectasis, SARS-CoV-2-typical vascular lesions, necrotizing bronchitis, or alveolar immune cell infiltrates (Fig. 2i). Only focal perivascular (8/12 in P.1 and 3/6 in P.4) and peribronchial (4/12 in P.1 and 0/6 in P.4) inflammatory infiltrates were observed (Fig. 2i). Focal to multifocal viral antigen was found in all groups except the washing group from passage 4, and this was associated with mild expansion of the pulmonary interstitium, mainly by macrophages (Fig. 2j).

The OTS-228 genome was sequenced from a nasal washing sample obtained from a P.4 hamster (tissue group) at 5 days post-inoculation (dpi) and compared to the OTS-228 reference sequence (Supplementary Figure 1a). This analysis revealed only two nonsynonymous mutations—one in ORF1a nsp3 (N1922Y) and one in the spike gene (S514Y) (Supplementary Figure 1b). All 325 modified leucine/serine codons (OTS codons) and the nsp1 modifications remained unchanged. In summary, these results demonstrate remarkable genomic stability of the modified genomic regions under forced in vivo transmission conditions, and that nasal wash samples from OTS-228-vaccinated hamsters were not able to induce infection after intranasal inoculation.

### In vitro passaging did not impair the safety profile or efficacy of OTS-228

**Fig. 3:**
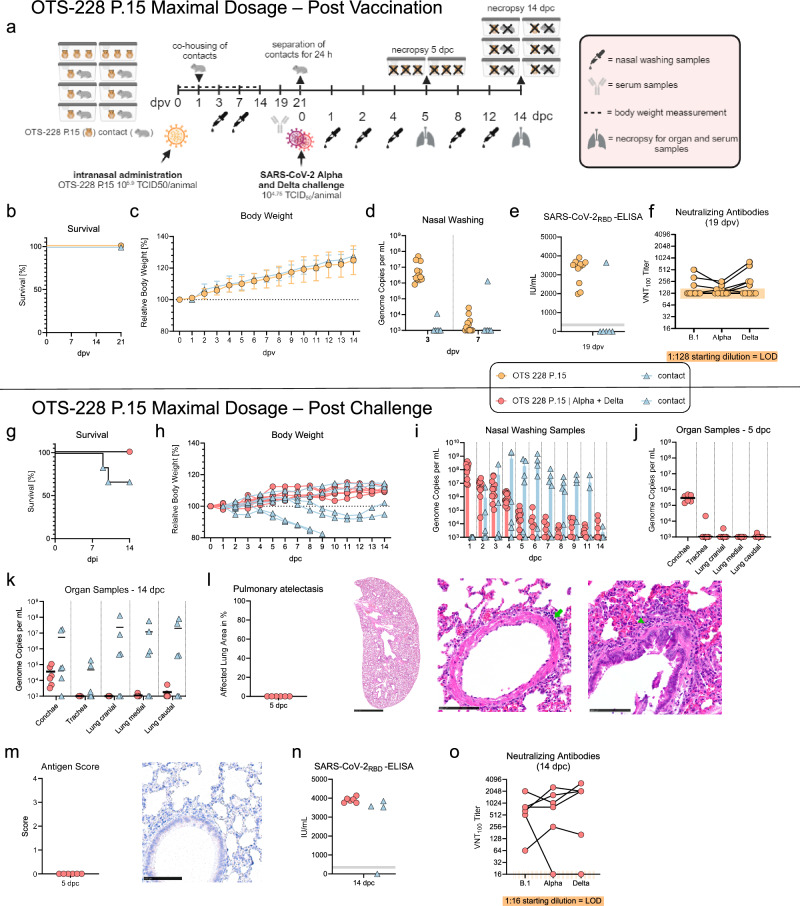
Intranasal vaccination of Syrian hamsters with a maximum dose of OTS-228 passage 15 and subsequent challenge infection. **a** Experimental setup: Intranasal vaccination of 12 index hamsters with 10^6.0^ TCID_50_/animal of OTS-228 P.15. Direct contact animals (n = 6) were co-housed 1 dpv. **b** Survival and (**c**) body weight were monitored daily, confirming no mortality or weight loss post-vaccination. **d** Shedding of OTS-228 vaccine virus genome was confirmed by RdRp (nsp12)-specific RT-qPCR of nasal wash samples, determining genome copies per mL (gc/mL). One of six direct contact animals tested positive on days 3 and 7. **e** Sera from 19 dpv were evaluated for SARS-CoV-2 RBD-specific antibodies by ELISA, confirming a humoral immune response in vaccinated animals and the genome-positive contact animal. **f** Virus neutralization test (VNT_100_): Some sera were tested for neutralizing antibodies against different VOCs (threshold > 1:128 starting dilution due to limited sample volume), showing positive results for WT (3/12), Alpha (4/12), and Delta (4/12). Three weeks post-vaccination, the vaccinated animals were challenged intranasally with a mixture of Alpha/Delta SARS-CoV-2 variants, and naïve direct contact animals were co-housed again one day post-challenge in a 1:1 setup. **g** Survival and (**h**) body weight were tracked until 14 dpc. None of the vaccinated animals showed mortality or weight loss, while two contact animals did not survived the infection. **i** Virus shedding: Viral genome copy numbers in nasal wash samples showed relatively high shedding on day 1 (mean 10^8.2 ^gc/mL) but a significant decline by day 5 (mean 10^4.7^ gc/mL, a 3017-fold reduction). **j** Viral genome load was also determined in organ samples at 5 dpc and (**k**) 14 dpc, with only residual viral genome detectable in lung samples at both time points. **l** Lung evaluation for pneumonia-related atelectasis at 5 dpc showed no significant damage (Hematoxylin-eosin stained whole lung slide images (bar 2.5 mm), with detailed images showing perivascular (green arrow) and peribronchial inflammatory infiltrates (green arrowhead) (bar 100 µm)), and (**m**) the absence of SARS-CoV-2 N-protein antigen indicated a high level of lung protection. **n** Humoral immune response: ELISA measurements confirmed transmission of infection in three of the four remaining contact animals, based on binding of the SARS-CoV-2 RBD-domain of the spike protein. **o** Neutralizing antibodies: At 14 dpc, the neutralizing humoral immune response, tested in a VNT_100_, confirmed high titers of biologically relevant cross-reacting neutralizing antibodies in the vaccinated hamsters.

To assess the genetic stability of vaccine candidate OTS-228 and the retention of its attenuation and protective efficacy, we intranasally inoculated Syrian hamsters (n = 12) with the maximum possible dose of OTS-228 after 15 in vitro passages (OTS-228 P.15). The in vitro passages were conducted in triplicate (rep.1–3) using Vero E6 cells. Sequencing results (Supplementary Figure 1c, *upper panel*) revealed a total of 15 cell culture-adaptive mutations (rep.1: 4, rep.2: 7, rep.3: 8), as previously reported^4^. The three replicates were pooled in equal amounts and used as inoculum (10^6.0^ TCID_50_/animal) (Fig. 3a). No mortality or weight loss was observed in either the vaccinated or contact hamsters (Fig. 3b, c). High genome copy numbers (10^5.9^–10^7.7^ gc/mL) were detected in the nasal washing samples of the vaccinated animals at 3 dpv, with only one of the six contact animals tested positive for viral RNA (10^4.1^ gc/mL) (Fig. 3d). Sequencing of the OTS-228 genome from the 7 dpv nasal washing sample of the contact animal revealed a change in 1 of the 325 modified Leucine/Serine OTS codons (S3732L) located in nsp6 (OTS fragment 5). Four additional consensus mutations were detected: one synonymous mutation (ORF1a: nucleotide exchange A7729C) and three nonsynonymous mutations (two in the spike gene: D253V, L1197P, and one in the M gene: T7I). Three of these mutations were present in the OTS-228 P.15 inoculum (Schön et al. ^4^, Supplementary Table 5), while two emerged during hamster infection (or were present at undetectable levels in the inoculum), and 12 cell culture-related mutations were lost (Supplementary Figure 1c, *lower panel*). Additionally, a new minor synonymous variant was found in the N gene (nucleotide exchange A29467G, 42% variant frequency) (Supplementary Figure 1c, *lower panel*). Seroconversion was confirmed in the vaccinated animals at 19 dpv by ELISA (Fig. 3e) and the development of neutralizing antibodies (Fig. 3f), with the only PCR-positive contact animal also showing seroconversion (Fig. 3e).

To evaluate the protective efficacy of OTS-228 P.15, we challenged the vaccinated hamsters with an equal mixture of SARS-CoV-2 Alpha and Delta variants (in total 10^4.8^ TCID_50_/animal) (Fig. 3a). The contact hamsters, which were introduced 24 h after vaccination, were separated again for 24 hours just before challenge infection (Fig. 3a). Post-challenge, none of the vaccinated animals experienced mortality or weight loss (Fig. 3g, h). However, two contact animals started losing weight from 4 days post-challenge (dpc) and succumbed to disease at 9 and 10 dpc (Fig. 3g), while two others lost weight between 8 and 9 dpc. The remaining two contact animals, including the one that tested positive for OTS-228 P.15 genome and seroconversion prior to challenge infection, did not lose weight (Fig. 3h).

All challenged animals tested positive for challenge virus genome in nasal wash samples, with a gradual decrease over time (Fig. 3i). Three contact animals tested positive at 2 dpc, one day post co-housing, while at later time points 5 out of 6 were tested positive (Fig. 3i). Evaluation of upper and lower respiratory tract (URT and LRT) organ samples at 5 dpc showed full protection from lung infection and limited replication in the URT in vaccinated animals (Fig. 3j). No infectious virus was detectable for nasal washing samples 3 dpc (Supplementary Figure 4a) and organ samples 5 dpc (Supplementary Figure 4b) by in vitro titration. By 14 dpc, organ samples confirmed that four out of six contact animals had become infected with the challenge virus (Fig. 3k). The vaccinated animals showed only low viral genome loads in conchae samples, while lung samples tested almost negative (Fig. 3k). Histopathologic examination of the lungs at 5 dpc revealed that the SARS-CoV-2 Alpha/Delta challenge infection after OTS-228 P.15 vaccination did not lead to pneumonia-related atelectasis, SARS-CoV-2-typical vascular lesions, or necrotizing bronchitis. Multifocal perivascular and peribronchial inflammatory infiltrates were observed, likely remnants from virus clearance (Fig. 3l). No virus antigen was found in the alveolar or bronchial epithelium (Fig. 3m). The absence of pulmonary atelectasis and viral antigen at 5 dpc confirmed the high level of protection. Serological analysis also showed that one contact animal remained seronegative, indicating reduced transmission (Fig. 3n). Additionally, vaccinated and challenged animals exhibited high levels of neutralizing antibodies (Fig. 3o).

### Fifty percent protective dose of OTS-228 vaccination is below 100 TCID_50_ in the Syrian hamster model

**Fig. 4:**
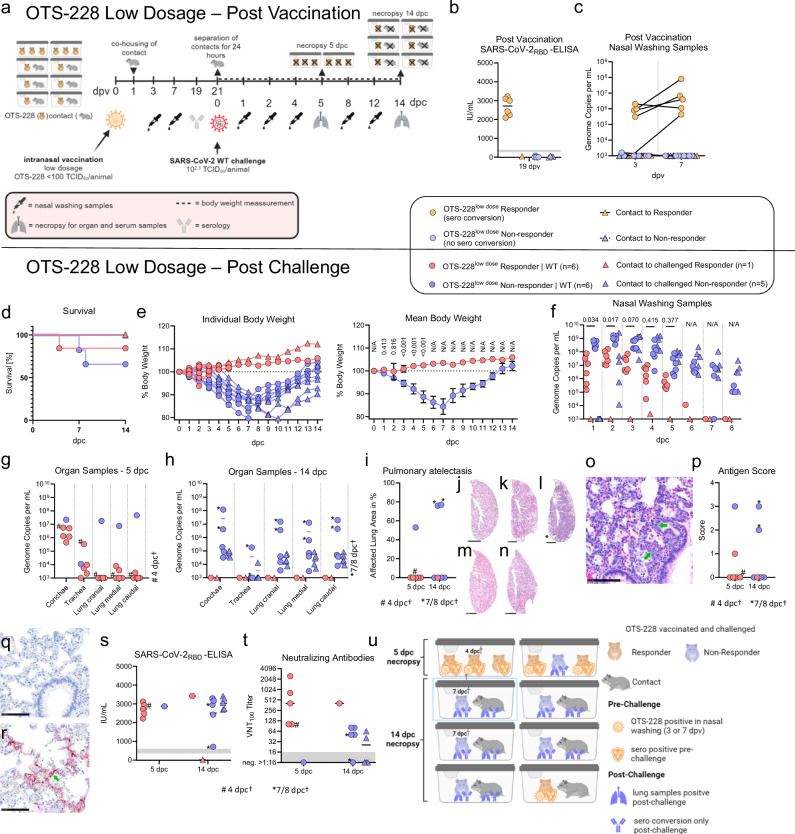
Intranasal vaccination of Syrian hamsters with low-dose OTS-228 and subsequent challenge infection. **a** Experimental Setup: Vaccination of 12 hamsters with low-dose OTS-228 (10¹–10² TCID_50_/animal). Six contact animals were co-housed one day post-vaccination to monitor potential transmission. **b** Antibody Response: Sera from 19 dpv were analyzed by RBD-specific ELISA. Six vaccinated hamsters showed a specific immune response (“responders”), while the others were designated “non-responders.” **c** Viral Genome Detection Post-Vaccination: Viral genome copies in nasal wash samples were quantified by RT-qPCR (orf1ab-specific), confirming virus presence in five of 12 vaccinated animals. **d** Survival Post-Challenge: Vaccinated animals were challenged with SARS-CoV-2 WT at day 21 post-vaccination. One responder died during sampling, while two non-responders succumbed to infection by day 7. **e** Body Weight Changes Post-Challenge: Responders maintained body weight, while non-responders exhibited significant weight loss by day 5 post-challenge. Statistical analysis was performed using a mixed-effects model with Geisser-Greenhouse correction and Sidak’s multiple comparisons test, with individual variances computed for each comparison between responder and non-responder groups. **f** Viral Shedding Post-Challenge: RT-qPCR analysis of nasal wash samples revealed significantly lower viral genome copies in responders compared to non-responders. Statistical analysis was performed using a mixed-effects model with Geisser-Greenhouse correction and Tukey´s multiple comparisons test, with individual variances computed for each comparison between responder and non-responder groups. **g, h** Viral Genome in Respiratory Organs: At 5 dpc, lung samples from non-responders contained high viral loads, while responders showed minimal virus. By 14 dpc, viral genomes were still detectable in non-responders but not in responders. **i** Histopathology, pneumonia-related atelectasis given in % affected area. Representative hematoxylin-eosin stained lung sections (bar 2.5 mm) are shown: (**j**) Lack of atelectasis in all responders, (**k**) moderate atelectasis (53%) in the non-responder at 5 dpc, (**l**) severe atelectasis (76–77%) in the two non-responding hamsters of 7/8 dpc and (**m**) lack of atelectasis for responders and (**n**) non-responders at 14 dpc. **o** Moderate hyperplasia and hypertrophy of type II pneumocytes (green arrow) for the non-responders at 14 dpc, bar 100 µm. **p–r** Antigen Detection: Immunohistochemistry revealed (**q**) minimal viral antigen in one of five responders (5 dpc) but (**r**) extensive antigen presence in three of six non-responders examined at 5/7/8 dpc, particularly in type I pneumocytes. **s**, **t** Seroconversion and Neutralizing Antibodies: All non-vaccinated hamsters seroconverted post-challenge, while neutralizing antibodies were present in responders, correlating with protection against lung infection. **u** Overall Conclusion: OTS-228 vaccination led to a robust humoral immune response in responders, correlating with protection against severe lung infection and virus replication. Non-responders experienced more severe outcomes, with higher viral loads and lung damage.

To evaluate whether a low dosage of OTS-228 vaccination is sufficient to elicit a broad immune response and provide protection against viral challenge infection, we intranasally vaccinated Syrian hamsters (n = 12) with a very low dose of OTS-228, using less than 100 TCID_50_ per animal in a single vaccine shot. At 21 days post-vaccination, we challenged the vaccinated animals with SARS-CoV-2 WT (Fig. 4a).

Following the low-dose vaccination, 6 out of 12 vaccinated hamsters showed positive reactions for SARS-CoV-2-specific antibodies at 19 dpv. The remaining six animals did not seroconvert. The seroconverted animals were subsequently designated as “responders,” while the six animals without seroconversion were designated as “non-responders” (Fig. 4b). Correspondingly, five of the responder hamsters were tested positive for the vaccine genome in nasal wash samples at 7 dpv (Fig. 4c).

After challenge infection with SARS-CoV-2 WT, the six responder hamsters did not exhibit weight loss. However, one animal died unrelated to the challenge infection under short-term anesthesia during the sampling procedure. The histological examination of this animal confirmed that there is no evidence of a correlation with the infection (Supplementary Figure 2). In addition, two of the non-responders succumbing to the infection at 7 dpc or reaching humane endpoint criteria by 8 dpc (Fig. 4d). In contrast, the six non-responders lost more than 10% of their body weight with in the first 5 dpc, which is significant (*p* < 0.001) more than the responders (Fig. 4e). In addition, the contact animals housed with the non-responders began to lose body weight from 1 dpc, whereas the single contact animal housed with the responder did not (Fig. 4d, e).

Virus shedding was significantly lower after challenge infection (*p* < 0.034, 1 dpc) in samples from the responder animals compared to the non-responders and only the contact animals to the non-responders began to shed from 2 dpc on (Fig. 4f). Furthermore, a high level of lung protection was observed in the responder animals, evidenced by the low abundance of viral genome in lung samples (Fig. 4g, h). Infectious virus was detected in nasal wash samples at 3 days post-challenge (dpc) in one of six responders, two of six non-responders, and three of five contact animals exposed to non-responders (Supplement Figure 5a). Organ samples were largely negative, with the exception of two low-level titers: one in a conchae sample from a responder and one in a caudal lung sample from a non-responder (Supplement Figure 5b).

Histopathological examination of the lungs of the responders showed no evidence of pneumonia-induced atelectasis or SARS-CoV-2-specific lesions (Fig. 4i, j, m). However, perivascular and peribronchial immune cell infiltrates and mild alveolar macrophage infiltration were observed in the majority of animals. In contrast, the non-responders exhibited moderate to severe atelectasis, SARS-CoV-2-characteristic vascular lesions, and necrotizing bronchitis during the acute phase of infection (Fig. 4k, l), accompanied by mild to moderate hyperplasia and hypertrophy of type II pneumocytes in the chronic, regenerative stage (Fig. 4n, o). Intralesional, multifocal to coalescing viral antigen was detected only in three out of six non-responders during the acute phase of infection, while focal sparse antigen was observed in one out of six responders. (Fig. 4p–r).

Challenge virus infection in the non-responders and virus transmission to the co-housed contact animals were confirmed via serological analysis. In contrast, transmission of the challenge virus to contact animals co-housed with the responders was blocked (Fig. 4s). The responders showed high neutralizing antibody titers as early as 5 dpc, while the non-responders remained negative (Fig. 4t). Although only one responder animal was used in a transmission setup, our data suggest that even a low-dose immunization can induce transmission-blocking immunity in principle (Fig. 4u).

Together, the data indicate a strong correlation between OTS-228 virus replication, seroconversion, and subsequent protection against viral challenge infection.

## Discussion

Live attenuated vaccines (LAVs) offer the full range of viral antigens by mimicking a natural infection in an attenuated form. This enables great potential to induce broad humoral and cellular immune responses both locally and systemically. Such a robust immune response could not only effectively prevent disease in vaccinated individuals, but also reducing transmission to unvaccinated individuals, potentially providing cross-protection against closely related pathogens^7–9^. Because LAVs mimic natural infections, they are typically administered at the site of infection and induce, in case of respiratory infections, local mucosal immune responses. This allows for non-invasive delivery methods such as nasal sprays or oral drops.

The SARS-CoV-2 LAV OTS-228 has shown high efficacy against several SARS-CoV-2 variants in preclinical studies using Syrian hamsters, an established and sensitive model for SARS-CoV-2 research^4^.

However, live vaccines can harbor some disadvantages, including the possibility of incomplete attenuation, the potential risk of reversion to virulence, and the risk of unwanted vaccine shedding. The most established live vaccines against diseases like measles^10^, mumps^11^, rubella^12^, yellow fever^13^ or poliomyelitis^14^ were generated traditionally by unspecific passaging in various cell cultures or embryonated chicken eggs. In contrast, OTS-228 was strategically designed by combining several attenuating genome modifications. For each of these modifications alone an attenuating effect is described: Nsp1 K164A, H165A mutations prevents host cell protein synthesis shut down^15,16^, spike cleavage site deletion is attenuating and prevent spreading^17^, large scale OTS-codon modifications increase chance of stop codon generation and reduces translational efficacy^18^ and the deletion of ORF6-8 prevents down regulation of the host immune responses^19–22^. There are many different experimental SARS-CoV-2 live vaccine concepts described so far, mostly focusing on just one or a few of these attenuation methods, not combining the full spectrum^3,23–27^. This combination greatly ensuring OTS-228´s robust attenuated phenotype and safety.

Our study showed that the maximum dose of the OTS-228 vaccine (10^6.1^ TCID_50_/hamster) was well tolerated, with no significant adverse effects such as weigth loss, pulmonary atelectasis, SARS-CoV-2 associated vascular lesions, or necrotizing bronchitis. Only a slight interstitial, perivascular and peribronchial infiltration of immune cells and some associated viral antigen was detected. In comparison, non-attenuated ancestral SARS-CoV-2 caused significant mortality, marked weight loss and severe pneumonia even at doses more than 1000 times lower^28–30^, highlighting the safety of OTS-228. In addition, our study revealed that even at this artificial maximum dose, the vaccine virus was not transmitted to naïve contact animals. This underscores its safety profile, especially considering that many studies have reported 100% transmission rates for unattenuated SARS-CoV-2 in Syrian hamsters in direct contact, even at much lower titers^31–34^.

A concern with LAVs is the potential for reversion to a more virulent form, as seen with the cold-adapted influenza virus vaccine^6^ and the oral poliovirus vaccine (OPV)^5^. To address this potential concern, we extensively tested OTS-228 for genomic stability, focusing on the integrity of the introduced attenuating modifications. Therefore, we passaged OTS-228 in vivo using the hamster model. Notably, passage was only successful using homogenized nasal tissue samples; nasal washing samples were not infectious to naïve hamsters. This supporting the non-transmissible nature of OTS-228 under direct contact conditions as previously reported^4^. It has been reported that OTS-228^4^, like other modified and unmodified SARS-CoV-2 strains, does not transmit in vivo when the spike S1/S2 PCS is removed^17,34,35^. This indicates that the PCS is critical for both virulence and transmission.

Sequencing of the viral genome obtained from homogenized nasal conchae tissue after four passages in vivo in hamsters revealed only two non-synonymous mutations - one in nsp3 and one in the spike protein - outside the altered genomic regions, suggesting no increased virulence. The integrity of the sequence-based attenuation was further confirmed when we vaccinated hamsters with the highest possible dose (10^6.0^ TCID_50_/hamster) of a 15-times in-vitro-passaged OTS-228. Despite some accumulated mutations that are expected in cell culture, this strain retained its fully attenuated phenotype in vivo. Unlike other LAVs, where reversion to virulence can occur with only a few point mutations^5,6^, OTS-228’s attenuation relies on several mechanisms including numerous OTS-codons, point mutations (nsp1), deletion of specific domains (spike PCS), and full-gene deletions (ORFs 6-8), making full reversion to virulence highly unlikely.

However, one of six serologically naïve direct-contact animals picked up OTS-228 P.15 shortly after co-housing with a vaccinated animal, and nasal washing samples at 3 dpv (2 days post co-housing) tested positive for the vaccine genome. Genome analysis revealed no major changes; all mutations present at a frequency greater than 50% were already present in the inoculum. This transmission was likely due to residual high-titer inoculum taken up immediately after co-housing. This is supported by the in vivo passaging results, which clearly showed that nasal washing samples from OTS-228-infected hamsters could not cause infection after direct nasal inoculation of hamster. Nevertheless, transmission in 1/6 cases is rare, in a direct contact setup, compared to the 100% transmission rate for unmodified SARS-CoV-2 in naïve hamsters^4,31–34,36^. Despite the fact that our direct contact setup does not correspond to the situation in the human population, where mainly airborne transmission plays a role, it has been demonstrated in hamster studies that transmission efficacy is comparable in both direct contact and airborne experimental setups^31,37,38^. Consequently, it is hypothesized that, if transmission of OTS-228 occurs at all, it should be also minimal under airborne conditions in the human population.

OTS-228 maintained its genomic integrity and attenuated phenotype without reversion to virulence, as confirmed by deep-sequencing. The safety and efficacy of the vaccine were unaffected by a few non-synonymous mutations. The extensive point mutations and deletions in the OTS-228 genome make reversion to virulence unlikely, requiring cumulative mutations and recombination events, that did not occur under experimental conditions^34^. OTS-228 also demonstrated efficacy at low doses, which is important for practical vaccination strategies. When hamsters were vaccinated with less than 100 TCID_50_ per animal, 50% showed efficient immune responses and protection against wild-type SARS-CoV-2. Responders to the low-dose immunization showed similar protection to those receiving higher doses. This demonstrates one of the advantages of LAVs: even small amounts of the vaccine can be effective, potentially reducing production costs.

In order to make a valid comparison between the efficacy of OTS-228 and other live attenuated or mRNA-based SARS-CoV-2 vaccines, the present study has adopted a established interval of 21 days between immunization and challenge in preclinical animal models^3,34,39–44^. Moreover, in the context of a pandemic, as evidenced by the experience with SARS-CoV-2, there is an imperative for rapid development and adaptation of new vaccines. This constitutes a further rationale for choosing a short interval and single-dose immunization for our trial. As demonstrated by both previous^3^ and current data, OTS-228 induces high titres of neutralizing antibodies 14 days after vaccination, with the majority of the vaccine virus being cleared by this time. Following a natural infection with SARS-CoV-2, seroconversion occurs within the first two weeks^45,46^ and the peak of neutralizing antibodies is observed after three to four weeks post onset of symptoms^45,47–49^. It can therefore be concluded that, at the time of challenge 21 days after vaccination with a live attenuated vaccine, the humoral immune response is already well developed. Further, OTS-228 elicited SARS-CoV-2-specific IgA antibodies in nasal and lung tissues just 5 days post infection^4^. However, it should be noted that there is currently no evidence to prove that these are the more potent dimeric secretory IgA antibodies. Further investigations are required to clarify this. Nevertheless, the potential for innate immune effects, such as viral interference in influencing protective immunity after 21 days, cannot be completely excluded. Longer intervals than those used in this study are needed to investigate cellular and long-term immunity.

The objective of this study was to enhance the characterization of OTS-228 and to demonstrate its efficacy. The study thus challenged against Alpha and Delta variants, which present the most severe challenge in the hamster model, rather than the more recent Omicron variants, against which hamsters already show reduced pathogenicity^50^. In a previous study, we have shown that OTS-228 is also capable of mediating cross-protective immunity against more recent variants of SARS-CoV-2 including Omicron BA.2, BA.5 or XBB.1.5 challenge^4^. In contrast to the challenge with ancestral SARS-CoV-2, however, transmission could not be completely prevented after challenge with the more recent variants.

A limitation of our studies to date is the lack of evidence demonstrating the impact of pre-existing immunity on the efficacy of OTS-228 and the advantages over mRNA vaccines. However, it has been shown that the live attenuated SARS-CoV-2 vaccine sCPD9 successfully reinfects pre-immunized hamsters after repeated intranasal inoculation and that immunity increases after the second dose. Furthermore, sCPD9 has been shown to provide better protection against SARS-CoV-2 infection and was significantly more effective in reducing transmission compared to mRNA vaccines^3,40^. These results suggest that a prior SARS-CoV-2 infection does not compromise the efficacy of LAV and that significant advantages over previous vaccines can be anticipated. In conclusion, we found OTS-228 to be safe and effective, with no inter-animal transmission, with genetic stability, and immunogenicity, while demonstrating a low protective dose 50 (PD_50_). These results highlight the potential of LAV OTS-228 for effective use in the prevention of COVID-19 and underscore its suitability for further clinical trials.

## Methods

### Hamster experiments

All experiments were conducted under BSL-3 conditions at the Friedrich-Loeffler-Institut, Germany, using specific pathogen free (SPF) male Syrian hamsters (*Mesocricetus auratus*) of five to nine weeks of age (Janvier labs). For inoculation (vaccination and challenge) a micropipette was used to administer a volume of 35 µl each into both nostrils, for the high dosage experiment 50 µl per nostril were used. Each inoculum was back-titrated on Vero E6 cells to confirm the tissue culture infectious dose 50% (TCID50) per hamster. To quantify virus shedding, each nostril was washed by 200 µl PBS using a micropipette and the reflux was collected in a safe lock sample tube. Inoculation and nasal washing were performed under open-drop isoflurane anesthesia leading to short term unconscious of less than a minute, reducing the overall stress for the animals. Body weight was determined by putting the hamsters on a fine scale with a d-value of 0.1 g. Physical well-being was assessed on a daily basis by qualified personnel through visual inspection. Body weight loss of equal or more than 20% post inoculation was defined as human endpoint. At distinct timepoints, animals were euthanized after isoflurane overdose by combining bleeding to death with severing of the spinal cord in the area of the occiput and upper respiratory (conchae nasalis and trachea) and lower respiratory tract samples (lung cranial, medial, caudal) of about 0,1 cm3 were taken to examine virus genome loads in these organ samples. Organ samples were homogenized in a 1 mL mixture composed of equal volumes of Hank’s balanced salts MEM and Earle’s balanced salts MEM with 1% penicillin–streptomycin at 300 Hz for 2 min using a Tissuelyser II (Qiagen) and were then centrifuged to clarify the supernatant. Beside blood collection at end timepoints, serological follow-up samples were taken by punctuation of the saphenous vein under isoflurane anesthesia administered via a face mask. A calibrated vaporizer was used to deliver isoflurane at a concentration of 2% (v/v) in oxygen. Serum, nasal washes and organ samples were stored at -80 °C. All hamster experiments were reviewed and approved by the ethics committee of the State Office for Agriculture, Food Safety, and Fisheries of Mecklenburg–Western Pomerania (LALLF M-V), and gained governmental approval under registration numbers LVL MV TSD/7221.3-1-001/22, 7221.3-2-005/21 and 7221.3-1-040/22.

### Experimental setup: Maximal dose experiment

The highest possible dose of OTS-228 (10^6.1^ TCID_50_/hamster) was used to intranasally inoculate 14 male Syrian hamsters of 5 weeks of age. One day post inoculation three naïve contact animals were co-housed in a 3:1 setup.

### Experimental setup: In vivo passage stability experiment

Initially, hamsters (n = 6) were intranasally inoculated with cell culture derived OTS-228 (10^3.8^ TCID_50_/hamster). Direct naïve contact animals were added in a 1:1 setup (3 inoculated with 3 contact) after 24 h. On 3 dpv, nasal washing samples were obtained and on 5 dpv, hamsters were euthanized to harvest conchae and lung samples (passage 1). For the next passage all nasal washing samples were pooled and used as inoculum for 3 hamsters (washing group), while another 3 hamsters were inoculated by conchae sample pool (tissue group). Again, nasal washing (3 dpv) and tissue samples (5 dpv) were obtained from both groups and tested by sensitive SARS-CoV-2 RT-qPCR (passage 2). The nasal washing sample with most viral genome of the tissue group served as inoculum for the next wash group passage, and a pool out of the two conchae samples with most abundant viral genome copies served as inoculum for the tissue group. Nasal washing and tissue samples were collected as before, with an additional washing sample at 5 dpv (passage 3). Respective nasal washing and conchae samples from the tissue group tested positive for viral genome served as inoculum for the next passage (passage 4).

### Experimental setup: Safety and efficacy of 15th in vitro passaged OTS-228

OTS-228 was passaged 15-times in triplicates on Vero E6 cells. The generated OTS-228 P.15 was used to intranasally vaccinate Syrian hamsters (n = 12) with a dosage of 10^6.0^ TCID_50_/hamster. One day post inoculation, six naïve direct contact animals were co-housed in a 1:1 ratio. Animals were challenged at 21 dpv using an equal mixture of SARS-CoV-2 Alpha (EPI_ISL_751799) and Delta (PQ136912) of 10^4,8^ TCID_50_/hamster. Just before challenge, the direct contact animals were separated for 24 h.

### Experimental setup: Low-dosage experiment

OTS-228 inoculum was diluted to a titer of less than 100 TCID_50_ per hamster (n = 12), back titration resulted a titer of 36.9 TCID_50_ per hamster, and then used for an intranasally single-shot vaccination. On 19 dpv serum samples were obtained and humoral immune response was determined by a specific SARS-CoV-2 RBD-ELISA, allowing discrimination between vaccine responder and non-responder. At 21 dpv the vaccinated animals were challenged with 10^2.3^ TCID_50_ of SARS-CoV-2 Wuhan. Following vaccination and challenge, direct contact animals (1:1 ratio) were separated for 24 h.

### Virus genome quantification

Nucleic acids were extracted from 100 μl of the nasal washing samples or 100 μl of organ/tissue sample supernatant using the NucleoMag Vet kit (Macherey Nagel). The detection of SARS-CoV-2-specific nucleic acids in the RdRp gene is performed using primers/probes recommended by the Pasteur Institute. Primers: SARS2-IP4-14059F: 5’—GGT AAC TGG TAT GAT TTC G -3’ (Forward), SARS2-IP4-14146R: 5’-CTG GTC AAG GTT AAT ATA GG -3’ (Reverse) and probe SARS2-IP4-14084: FAM: 5’-FAM- TCA TAC AAA CCA CGC CAG G -BHQ-3’. The primer/probe combination targets a region within the RNA encoding the RdRp protein: nucleotides 14080–14186 of the genomic sequence of the “Severe acute respiratory syndrome coronavirus 2”^51^. To verify successful RNA extraction, the housekeeping gene beta-actin is detected in addition in the respective samples using specific primers^52^. The primers and probe used for this purpose are as follows: ACT-1030-F: 5’-AGC GCA AGT ACT CCG TGT G -3’ (Forward) targeting nucleotides 1030 – 1047, ACT-1135-R: 5’-CGG ACT CAT CGT ACT CCT GCT T-3’ (Reverse) targeting nucleotides 1114–1135, ACT-1081-HEX: 5’-HEX-TCG CTG TCC ACC TTC CAG CAG ATG T-BHQ1-3’ (Probe). The detection of actin using the forward primer ACT-1030-F identifies both genomic DNA and RNA, confirming a successful sample collection and RNA isolation. The RT–qPCR reaction was prepared using the qScript XLT One-Step RT–qPCR ToughMix (QuantaBio) in a volume of 12.5 µl including 1 µl of the respective FAM mix and 2.5 µl of extracted RNA. The reaction was performed for 10 min at 50 °C for reverse transcription, 1 min at 95 °C for activation, and 42 cycles of 10 s at 95 °C for denaturation, 10 s at 60 °C for annealing and 20 s at 68 °C for elongation. Fluorescence was measured during the annealing phase. RT–qPCRs were performed on a BioRad real-time CFX96 detection system (Bio-Rad). A dilution row of a standard with known concentration determined by digital droplet PCR was carried along to calculate the viral genome copy number per mL.

### Virus titration

Virus titers were assessed by standard endpoint dilution assays on Vero E6 cells. In short, cells were seeded in a 96-well plate 1 day before the titration, to achieve an 80-90% confluent cell layer, and were then inoculated with 100 µl of a 10-fold serial dilution of the sample. Sixteen technical replicates were performed for each dilution step. Cells were then incubated for 72 h at 37 °C in a humidified incubator with 5% CO_2_. Virus titer was calculated using the Spearman Kärber method^53,54^.

### RBD-specific enzyme-linked adsorption assay (RBD-ELISA)

The ELISA was performed similarly to the method described in ref. ^55^, demonstrating high sensitivity and specificity. Binding plates (Greiner, Kremsmünster, Austria) were coated with 100 ng/well of SARS-CoV-2 RBD antigen overnight at 4 °C in 0.1 M carbonate buffer (1.59 g Na_2_CO_3_ and 2.93 g NaHCO_3_, in 1 L distilled water, pH 9.6). The plates were then blocked for 1 h at 37 °C using 5% skim milk in phosphate-buffered saline (PBS). Serum samples were diluted 1:100 in Tris-buffered saline with Tween 20 (TBST) and incubated on the coated wells for 1 h at 37 °C. A multi species conjugate (SBVMILK; obtained from ID Screen® Schmallenberg virus Milk Indirect ELISA; IDvet) diluted 1/80 was applied. Following the addition of tetramethylbenzidine (TMB) substrate (IDEXX), ELISA readings were taken at a wavelength of 450 nm using a Tecan instrument (Tecan Group Ltd, Männedorf, Switzerland), resulting in optical density (OD450nm) values. Between each step, the plates were washed three times with TBST. For each run, the same defined two replicates of negative (NC) and positive (PC) control sera were included.

### Virus neutralization test (VNT)

The in vitro assessment of the live virus neutralizing potential of serum samples was conducted on Vero E6 cells (Vero C1008) using 96-well plates (Greiner, Kremsmünster, Austria). Sera were diluted two-fold in triplicate and mixed with an equal volume of SARS-CoV-2, containing 100 TCID_50_ per well. After a 1-hour incubation of the sample-virus mixture at 37 °C, 100 µL of trypsin-treated cells (trypsinized cells from a confluent 75 cm² cell culture flask in 50 mL DMEM with 2% penicillin/streptomycin) were added to each well. After three days of incubation at 37 °C, the presence of cytopathic effect (CPE) was evaluated using a standard optical transmission microscope. The VNT titer was determined from three independent dilutions that completely prevented CPE in the culture. The virus neutralization titer was then calculated using the formula: (−log2) = a/b + c, where (a) is the number of cell culture wells without virus replication, (b) is the number of cell culture wells per sera dilution, and (c) is the -log2 of the pre-dilution of the serum/yolk sample. The final titer is reported as -log2 value of the final sample dilution that completely prevents CPE formation on Vero E6 cells infected with 100 TCID_50_ and given as virus neutralization titer 100 (VNT_100_).

### Sequencing

Animal samples were sequenced using a generic metagenomics sequencing workflow as described previously^56^. In brief, extracted RNA was reverse-transcribed into cDNA using the SuperScript IV First-Strand cDNA Synthesis System (Invitrogen, Germany) and the NEBNext Ultra II Non-Directional RNA Second Strand Synthesis Module (New England Biolabs, Frankfurt am Main, Germany). Ion Torrent–compatible sequencing libraries were prepared and sequenced with chemistry for 400 base pair reads on an Ion Torrent S5XL instrument (Thermo Fisher Scientific, Darmstadt, Germany). Genome sequences were generated using reference mapping analysis applying the Genome Sequencer software suite (version 2.6; Roche; default software settings for quality filtering and mapping) with OTS-228 as reference. Obtained sequences were compared with the reference (MAFFT v.7.38837), and single nucleotides variants were detected applying the variant analysis tool integrated in Geneious Prime 10.2.3 (Biomatters; https://www.geneious.com).

### Histopathological and immunohistochemical analysis

For histopathology, the left lung lobe was carefully removed, immersion-fixed in 10% neutral buffered formalin, embedded in paraffin, and 2-3-μm sections were stained with hematoxylin and eosin (HE). Consecutive sections were processed for immunohistochemistry (IHC) used according to standardized procedures of avidin-biotin-peroxidase complex (ABC)-method. In detail, endogenous peroxidase was quenched on dewaxed lung slides with 3% hydrogen peroxide in distilled water for 10 min at room temperature (RT). The antigen was subjected to heat retrieval in a pressure cooker for 20 min in 10 mM citrate buffer (pH 6). To block nonspecific antibody binding, the slides were incubated for 30 min at RT with goat normal serum, diluted in PBS (1:2). A primary antibody against the SARS-CoV nucleocapsid protein was applied overnight at 4 °C (Rockland, 200-401-A50, 1:3000), and the secondary antibody, a biotinylated goat anti-rabbit antibody, was applied for 30 min at RT (Vector Laboratories, Burlingame, CA, USA, 1:200). The color was developed by incubating the sections with an ABC solution (Vectastain Elite ABC Kit; Vector Laboratories), followed by exposure to a 3-amino-9-ethylcarbazole substrate (AEC Substrate Kit, abcam). The sections were counterstained with Mayer’s haematoxylin and cover slipped. As a negative control, consecutive sections were labeled with an irrelevant antibody (capsid protein of Rustrela virus, clone 2H11B1). In each run, an archived control slide from a SARS-CoV-2-infected Syrian hamster was included. All slides were scanned with a Hamamatsu S60 scanner and analyzed by a trained pathologist (TB) and reviewed by a certified pathologist (AB) in a blinded manner using NDPview.2 plus software (Version 2.8.24, Hamamatsu Photonics, K.K. Japan). The lung tissue was evaluated using a 500 × 500 μm grid, and the extent of pneumonia-associated consolidation was recorded as a percentage of the affected lung fields. Furthermore, the lung was examined for the presence of SARS-CoV-2-characteristic lesions described for hamsters. These included intra-alveolar, interstitial, peribronchial and perivascular inflammatory infiltrates, alveolar edema, necrosis of the bronchial epithelium, diffuse alveolar damage, vasculitis, activation of endothelium with immune cell rolling, as well as bronchial epithelial and pneumocyte type 2 hyperplasia. Following IHC, the distribution of virus antigen was graded on an ordinal scale. The scores were as follows: 0 = no antigen, 1 = focal, affected cells/tissue < 5% or up to 3 foci per tissue; 2 = multifocal, 6%–40% affected; 3 = coalescing, 41%–80% affected; 4 = diffuse, >80% affected. The target cell was identified based on its morphological characteristics.

### Statistics and visualization

Data visualization and statistical analysis were performed using GraphPad Prism 10.2.1 (GraphPad Software Inc., USA). Experimental setups were illustrated with BioRender.com.

## Supplementary information

Supplementary Figures

## Data Availability

All datasets generated and analyzed during this study are included in the manuscript and supplementary files. The original raw data can also be obtained from the corresponding authors upon request. Genome sequences generated within this study are accessible at NCBI under accession numbers PQ536473 and PQ536474.
