# Supplementary material for: Live attenuated SARS-CoV-2 vaccine OTS-228 demonstrates efficacy, safety, and stability in preclinical model
Source: NPJ Vaccines. 2025 May 23;10:104. doi: 10.1038/s41541-025-01165-2 (PMC12098885; doi:10.1038/s41541-025-01165-2)
Supplement: Supplementary file 1 — Supplementary Figures [file 41541_2025_1165_MOESM1_ESM.pdf]

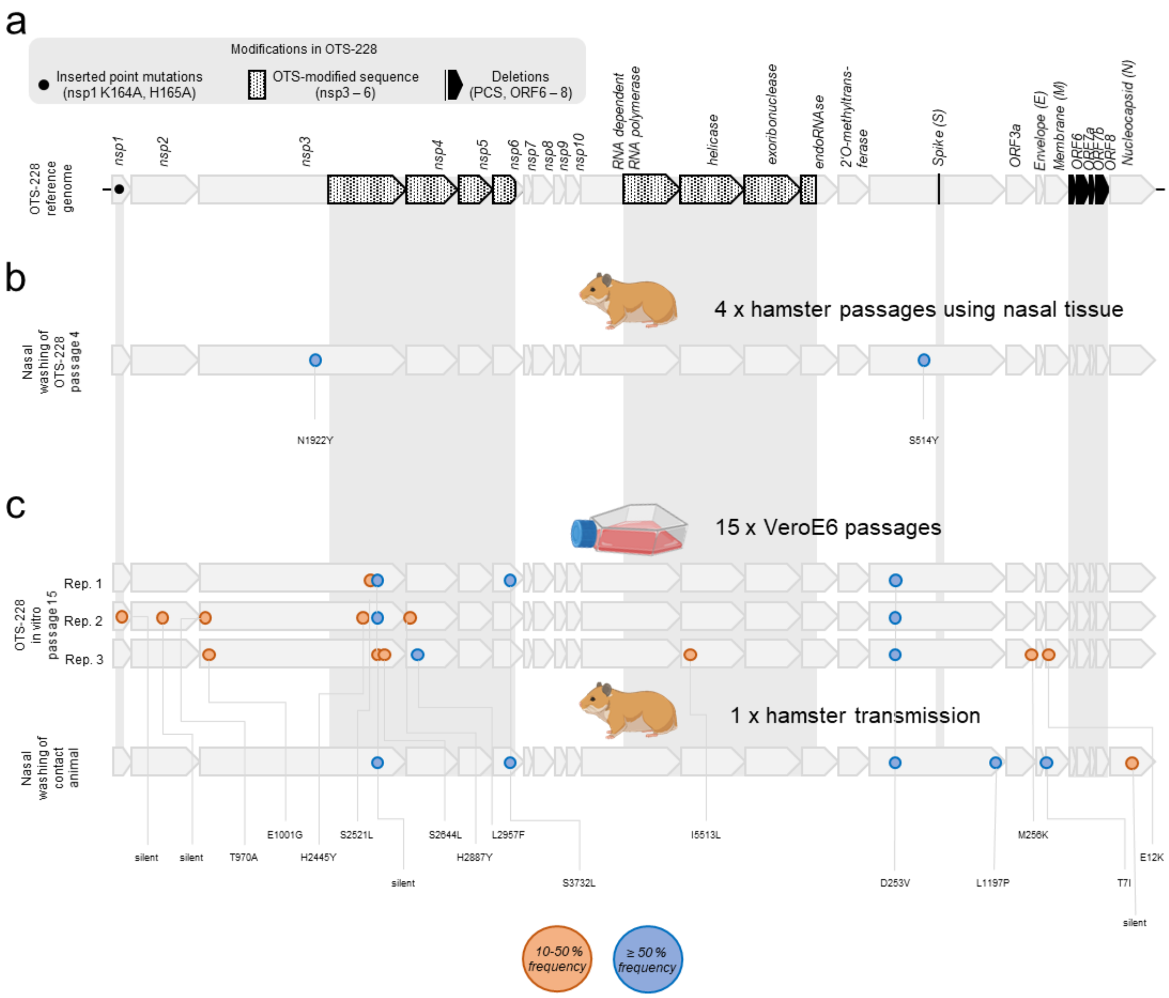

Supplementary Figure 1: Structure of OTS-228 and Deep Sequencing Analysis of *In Vitro* and *In Vivo* Samples. (a) Genomic structure of OTS-228, based on the ancestral SARS-CoV-2 sequence (MT108784.1). Attenuation is achieved through: i) point mutations nsp1 K164A and H165A, ii) OTS-codon modifications in ORF1ab, iii) PCS deletion, and iv) deletion of ORF6-8. (b) OTS-228 sequence from nasal washing samples after four passages in hamsters, each based on nasal tissues collected at 5 days post-inoculation (dpi) (accession number PQ536474). (c) OTS-228 sequence following 15 passages in Vero E6 cell culture across three biological replicates (Schön et al. 2024, Supplementary Table 5) [4]. These passages were pooled equally and used as the vaccine inoculum in a combined safety and efficacy study in hamsters. One day post-vaccination, six direct contact animals were co-housed in a 1:1 setup, creating six pairs for transmission evaluation. In one pair, OTS-228 was transmitted to the contact animal. OTS-228 was sequenced from nasal washings at 3 and 7 dpv (accession number PQ536473).

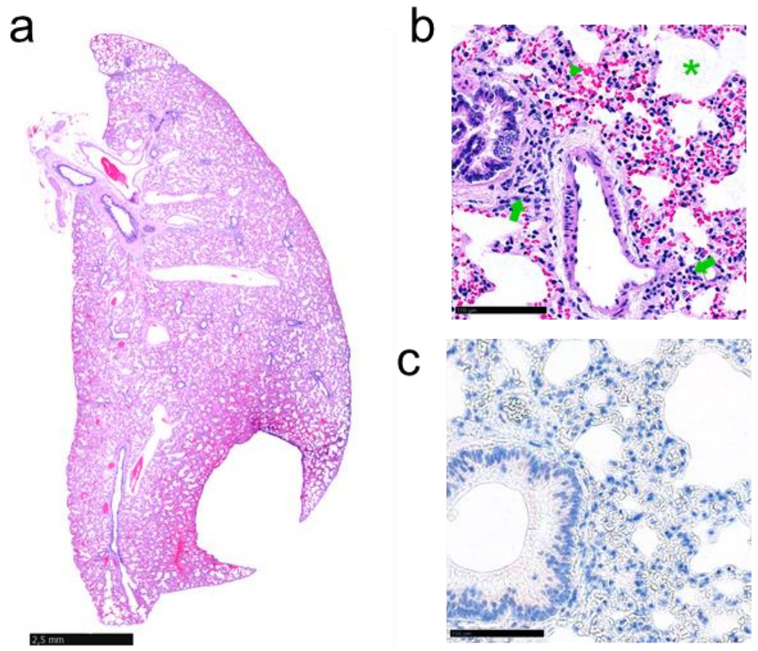

Supplementary Figure 2: Detailed Histopathology of the Lung in the Responding Hamster that Died 4 dpc. (a) Representative hematoxylin-eosin stained sections showing the absence of pneumonia-related atelectasis (bar = 2.5 mm). (b) Minimal perivascular and peribronchial immune cell infiltrations (green arrow), hyperemia (green arrowhead), and mild edema, but no alveolar inflammatory infiltrates (green asterisk) (bar = 100 µm). (c) Representative anti-SARS-CoV N-protein immunohistochemistry lung section illustrating the lack of antigen (bar = 100 µm).

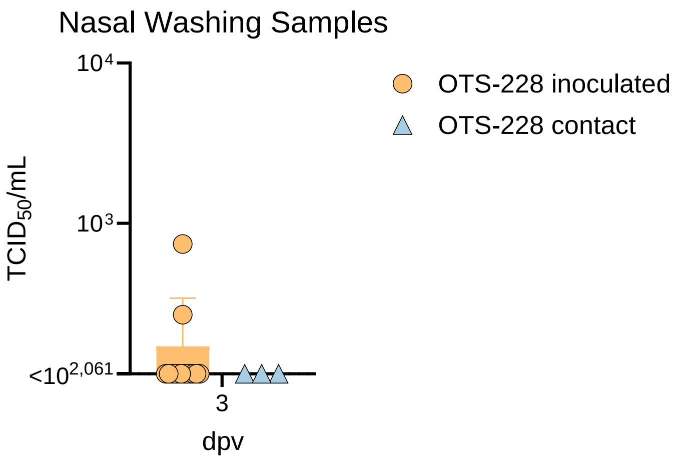

Supplementary Figure 3: In vitro titration of nasal washing samples 3 days post vaccination with OTS-228 maximum dose.

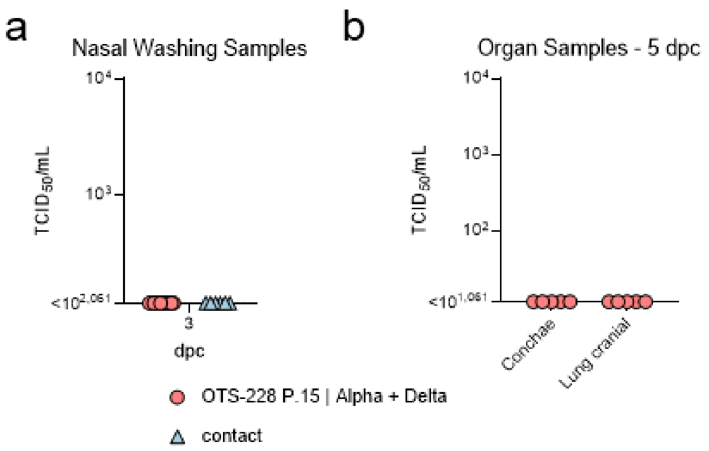

Supplementary Figure 4: In vitro titration of (a) nasal washing samples (3 dpc) and (b) organ samples 5 days post challenge infection with Alpha/Delta of maximal dose OTS-228 P.15 vaccinated animals.

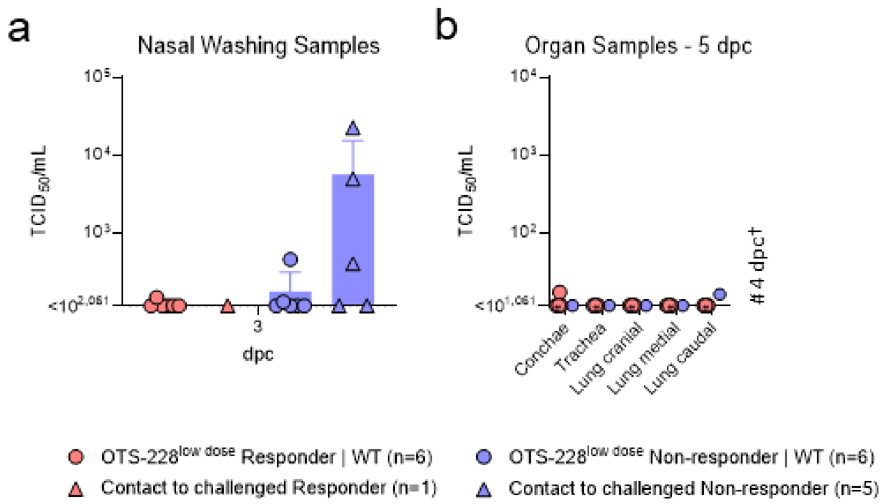

Supplementary Figure 5: In vitro titration of (a) nasal washing samples (3 dpc) and (b) organ samples 5 days post challenge infection with WT of low dose OTS-228 vaccinated animals.
